# Lateral size selection of liquid exfoliated hexagonal boron nitride nanosheets†

**DOI:** 10.1039/c7ra12872j

**Published:** 2018-02-05

**Authors:** Wei Gao, Yan Zhao, Hong Yin

**Affiliations:** State Key Lab of Superhard Materials, Jilin University Qianjin Street 2699 Changchun 130012 PR China hyin@jlu.edu.cn

## Abstract

Hexagonal boron nitride (h-BN) is of great importance in imaging, thermal and quantum applications in the mid-infrared regions (most of which are size related) for its natural hyperbolic properties. Liquid exfoliation is a promising production approach, however it is limited by the wide range of nanosheet length and thickness. Here we demonstrate a simple and effective method to sort the exfoliated nanosheets according to their lateral sizes. The process uses a combination of low-rate and high-rate configurations to separate the lateral lengths ranging from 1 to 3 μm. In contrast to Raman spectroscopy, infrared results exhibit intensive dependence on the nanosheet length, where the E_1u_ phonon frequency and intensity ratio of E_1u_ to A_2u_ modes are significantly sensitive to the nanosheet length owing to the large anisotropy between the in-plane and out-of-plane axes.

## Abstract 2

Hexagonal boron nitride (h-BN) is of great importance in imaging, thermal and quantum applications in the mid-infrared regions (most of which are size related) for its natural hyperbolic properties.

## Graphical abstract

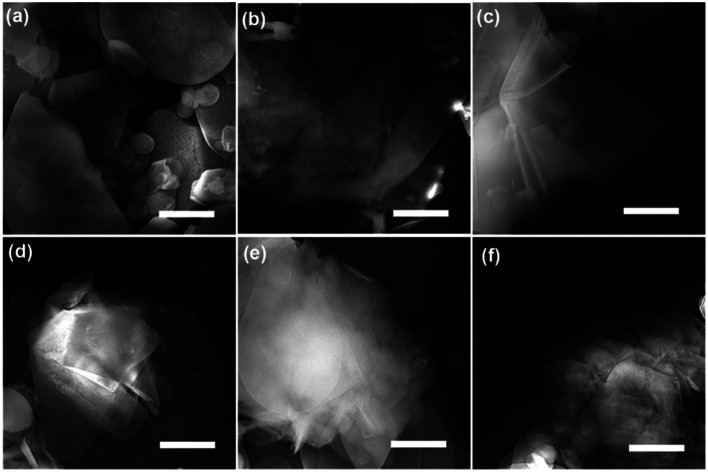

## 1. Introduction

Two-dimensional (2D) crystals exhibit exotic properties, arising from the unique structure of the individual atomic layers weakly coupled by van der Waals (vdW) interactions, that could be exploited for applications including electronics, optoelectronics, spintronics, chemical sensors, catalysis, energy storage, *etc.*^1–11^ Hexagonal boron nitride (h-BN) is one of the most representative layered vdW materials as it exhibits natural hyperbolic properties, in which the dielectric constants are the same in the basal plane but have opposite signs in the normal plane. Such properties of h-BN could inspire new imaging, thermal and quantum applications in the mid-infrared (mid-IR) wavelength region.^12–15^ In addition, this mid-IR spectral region is of extreme importance for it contains “fingerprints” of the most common molecular vibrations.

So far, various approaches have been proposed to fabrication of 2D nanomaterials, including bottom-up synthesis from an appropriate substrate,^8,15–18^ and top-down exfoliations based on either micromechanical method or chemical routes^2,19–21^ generally utilized to obtain layered crystals directly from their bulk counterpart. Large-scale exfoliation of layered materials in liquid phase is considered to be prospective in wide range of applications for high throughput and easy formation of novel hybrid and composite materials.^7,22–24^ However, one of the drawbacks in liquid exfoliation methods is the wide distribution in both longitudinal layer thickness and lateral nanosheet sizes ranging from tens of nanometers to a few microns, whereas many fundamental researches and applications of mentioned above depend sensitively on the size-related properties. For instance, it is of extreme importance of the lateral size for the nanosheets as fillers in polymer reinforcement.^25–30^ Besides, the behavior of phonon polaritons in h-BN could be governed by the crystal thickness and the launching distance from the edge.^11^

Atomic force microscopy (AFM), transmission electron microscopy (TEM) and scanning electron microscopy (SEM) are generally utilized to measure the nanosheet size by depositing on an appropriate substrate, however which can be very statistically and time consuming. Coleman's group has developed *in situ* measurement of size and thickness of liquid-exfoliated MoS_2_, WS_2_, MoSe_2_ and WSe_2_ nanosheets based on the effect of edges and quantum confinement on their corresponding optical spectra, whereas applicable only for the nanosheet size less than 300 nm.^20,31^ Moreover, a great deal of efforts on Raman spectra of D and G bands of carbon has been proposed to measure the lateral size and thickness of graphene and graphene oxide flakes indirectly.^32–34^ In spite of the similar structure to graphene, recently Raman signature and phonon dispersion of h-BN nanosheets (BNNSs) have been found not thickness-related,^35^ though it is widely used to characterize the properties of BNNSs.^36^ Thus, it is highly indispensible to search alternative methods to sort the lateral size and thickness of h-BN nanomaterials. Fourier transformed infrared (FTIR) is of more interest for two reststrahlen bands in the mid-IR region spanning the transverse (*ω*_TO_) and longitudinal (*ω*_LO_) phonon frequencies of the out-of-plane mode (parallel to *c* axis, *ω*_TO_ = 760, *ω*_LO_ = 825 cm^−1^) and the in-plane mode (perpendicular to *c* axis, *ω*_TO_ = 1370, *ω*_LO_ = 1614 cm^−1^) in h-BN system. Specifically, the layered nature of two-dimensional BN nanomaterials here facilitates the observation of phonon activities by varying the thickness *d* and the nanosheet length *L* of the samples.^11^

Here, we propose a simple and effective approach to select chemically exfoliated h-BN nanosheets according to their lateral size varying from 1 to 3 μm based on controlled centrifugation process. Both Raman and IR transmission measurements have been performed to probe the correlation of the phonon profile with the nanosheet length. We show that Raman-active E_2g_ mode is not susceptible enough to determine the lateral size of BNNSs. On the contrary, the infrared-active in-plane E_1u_ phonon peak and the intensity ratio of E_1u_ and A_2u_ phonon modes vary intimately with the nanosheet length thanks to the strong underlying anisotropy and availability of the specimens with great control of size. We believe that this perspective of natural hyperbolic vdW crystals will shed light on the future applications in the areas of far-field imaging and focusing with subdiffraction resolution, broadband negative refraction, and superb control over light emission.

## 2. Experimental

### 2.1. Preparation of BNNSs

High quality h-BN nanosheets were directly exfoliated from h-BN bulk powder (99.5%, particle size 1–5 mm, Alfa Aesar) using acid solution. Briefly, a mixture of h-BN bulk powder (1 g) and potassium permanganate (KMnO_4_, Beijing Reagent, 6 g) with mass ratio of 1 : 6 was added into an acid mixture of sulphuric acid (98% w/w, Beijing Reagent) and phosphoric acid (85% w/w, Beijing Reagent) with volume ratio of 8 : 1 for 135 ml. The obtained solution was then constantly stirred at 75 °C for 12 hours. Differently to previous report,^19^ a frozen mixture of 6 ml hydrogen peroxide (H_2_O_2_, 30% w/w, Beijing Reagent) and 120 ml de-ionized water (DI water) was subsequently poured into the solution, then lowering the solution to the temperature of ice and water mixture under constantly stirring for another one hour. The resultant suspension was naturally back to room temperature, subject to a series of washing steps with DI water, ethanol and HCl until pH > 3. Finally, the supernatant solution is dried to obtain the mono- and few-layer BNNSs. Centrifugation (Avanti-J-26XPI, Beckman Coulter Inc.) was carried out for 45 min in all high-rate cases. Specially in the present work, we use 16 000, 18 000, and 20 000 rpm for the high-rate centrifugation, which corresponds to centrifugal force of 30 966, 39 191 and 48 384 × *g*.

### 2.2. Characterization

AFM (Bruker DI) and SEM (Philips FEI Quanta Magellan 400) were utilized to investigate the BNNSs. The microstructure and morphology of the as-grown BNNS were characterized using a SEM, an X-ray diffractometer (XRD, Bruker D8 using a Cu Kα radiation), FTIR (Nicolet Avatar 370) and a high resolution TEM (JEOL JEM-2200FS). Raman spectra were acquired at room temperature using a Raman spectrometry system (Jobin-Yvon T64000) yielding a high spectral resolution less than 0.15 cm^−1^ and a 514 nm laser (Lexel SHG-95) as an excitation source.

## 3. Results and discussion

The high-quality atomically thin h-BN nanosheets were produced by liquid exfoliation methods which generally results in mono- and few-atomic layers with lateral sizes ranging in several tens of nanometer to several micrometers. In order to screen different sizes of these exfoliated nanosheets, we propose a simple centrifugation technique based on the band sedimentation. Similar approaches have previously allow to sort carbon nanotubes,^37^ graphene oxide nanosheets^38^ and nanoparticles.^39^ A stock dispersion of exfoliated h-BN nanosheets was subjected firstly to a low-rate centrifugation (typically 3000 and 5000 rpm for 20 min) using a normal bench-top centrifuge. Such pre-centrifugation leads to the spreading of the mixed materials according to the mass difference from top to bottom in which the sediment generally contains non-exfoliated BN crystals and big flakes. Therefore the supernatant, which is measured to contain multilayered flakes, subsequently undergoes a further high-rate centrifugation using a refrigerated supercentrifuge speeding from 16 000, 18 000 to 20 000 rpm. The resultant 80% supernatant was pipetted off and the as-obtained bottom fraction has been analyzed by Raman spectroscopy, FTIR and XRD to extract a size-related correlation.

**Fig. 1:**
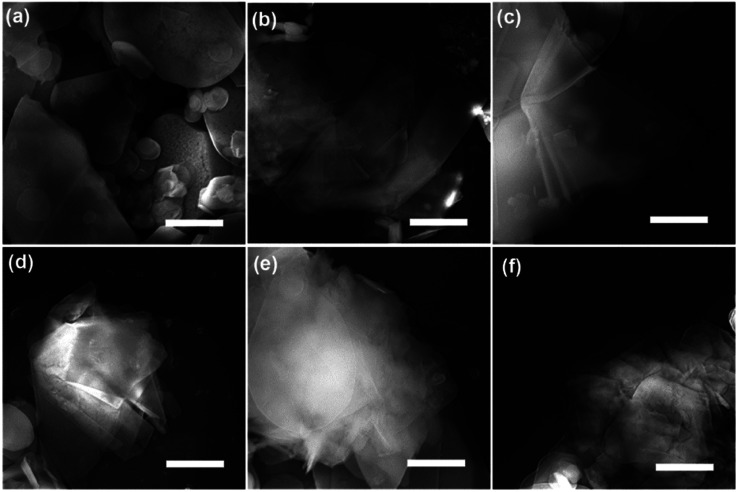
Typical exfoliated h-BN nanosheets after centrifugation process with size selection. SEM images of the pristine h-BN powder (a), exfoliated BNNSs by pre-centrifugation at 3000 rpm following with super-centrifugation at 16 000, 18 000 and 20 000 rpm (b–d) and exfoliated BNNSs by pre-centrifugation at 5000 rpm following with super-centrifugation at 18 000 and 20 000 rpm (e and f). The scale bar represents 1 μm.

**Fig. 2:**
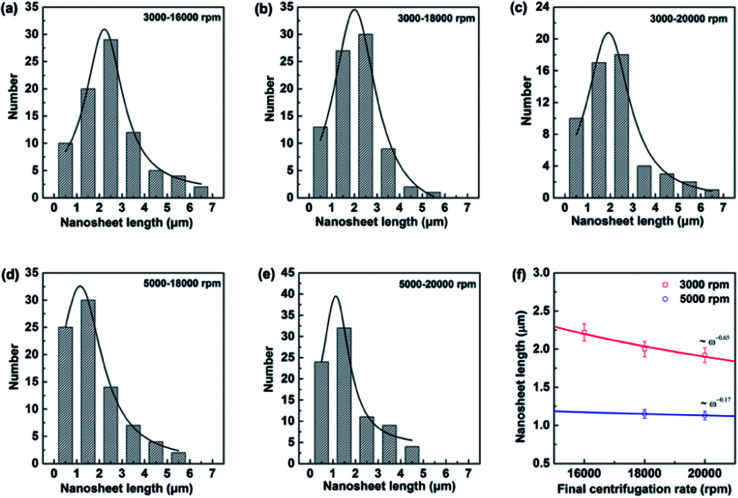
Histograms of nanosheet length distributions for different centrifugation processes (a–e). (f) Mean nanosheet length as estimated from (a–e) as a function of final centrifugation rate. The red and blue solid lines indicate an empirical scaling behavior of *ω*^−0.65^ and *ω*^−0.17^ for the nanosheets undergoing pre-centrifugation of 3000 and 5000 rpm respectively.

SEM and TEM analysis showed a range of as-obtained nanosheets as well as the pristine h-BN powder. High magnification SEM images in Fig. 1a–f display the pristine h-BN powder and exfoliated BNNSs bearing different high-rate centrifugation process. The bulk BN powders consist of irregular shapes and thick flakes with lateral size ranging from several to tens of micrometers (mean lateral length 〈*L*〉 ∼ 5 μm in present case). It is clearly observed that the morphology of BNNSs is distinct from that of their bulk counterpart where the longitudinal thickness is so significantly reduced that the layer is close to transparent. The flakes centrifuged at 20 000 rpm are much smaller than the 16 000 rpm ones. In order to understand the centrifugation process, the thickness and length of BNNSs are statistically studied comprehensively. As mentioned that 3000 and 5000 rpm was utilized to remove the big flakes prior to the high-rate centrifugation, two sets of statistical data are present with respect to the high-rate centrifugation from 16 000 to 20 000 rpm. Most of the BNNSs after centrifuge consisted of thin sheets with less than six layers (*i.e.*, were less than 2 nm thick) irrelevant of the pre-centrifugation (see TEM analysis in ESI†), whereas the length of nanosheets behaves differently. Indeed, liquid exfoliation method will result in the lateral nanosheet length of BNNSs ranging typically from several hundred of nanometer to several micrometers.^19,25,26^ Since size selection is possibly achieved by chromatography in limited quantities of materials, for practical meaning the controlled centrifugation has been adopted. The lateral size (the nanosheet length, *L*) is determined over 80 sheets for each high-rate centrifugation. Assuming Gaussian distributions, the resulting histograms allow us to extract average values for these data as displayed in Fig. 2a–e. The mean nanosheet length 〈*L*〉 is estimated to be 2.22, 2.0 and 1.92 μm for the centrifugation rate *ω* at 16 000, 18 000 and 20 000 rpm after the pre-centrifugation of 3000 rpm, and 1.15 and 1.13 μm for the centrifugation rate *ω* at 18 000 and 20 000 rpm after pre-centrifugation of 5000 rpm, respectively. The correlation between the centrifugation rate and the resulting nanosheet length is plotted in Fig. 2f. It clearly demonstrates that the nanosheet length *L* decreases as the final centrifugation rate (rpm) increases, strongly indicating that centrifugation at a high rate leads to the separation of small flakes from large flakes in the sediment. Empirically, these two quantities obey a power law as *ω*^−0.65^ for pre-using low centrifugation rate of 3000 rpm and *ω*^−0.17^ for 5000 rpm. It is worthy mentioning that the former relationship is consistent with those in other two-dimensional (2D) atomic materials.^33^

**Fig. 3:**
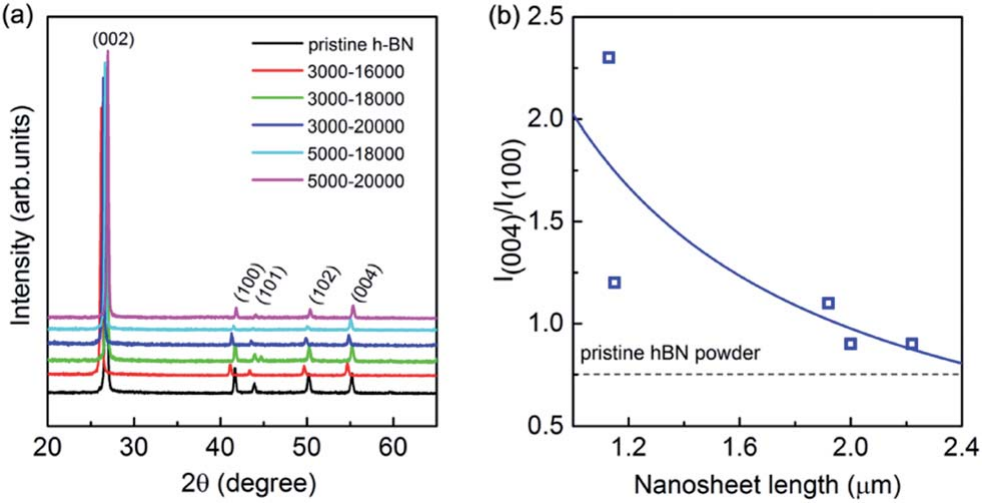
(a) Comparative XRD patterns of the bulk h-BN powders and the exfoliated BNNSs after centrifugation process. (b) Intensity ratio of XRD (004)/(100) patterns measured on exfoliated BNNSs from size selected dispersions as a function of lateral nanosheet length. The solid line is to guide the readers' eyes and the dash line is added as a reference of the typical pristine bulk h-BN powder.

XRD measurements were executed to verify the crystallinity of the exfoliated h-BN by drop cast onto a SiO_2_/Si substrate. The comparative XRD patterns are present in Fig. 3a, in which a bulk h-BN sample is added. From the Joint Committee on Powder Diffraction Standards (card number 85-1068) for h-BN powder of a lattice constants of *a* = *b* = 2.504 Å and *c* = 6.656 Å, all the diffraction peaks are readily indexed to be (002), (100), (101), (102) and (004), where the (004) is parallel to the (002) plane. The exfoliated sample exhibits considerable changes comparing to its bulk counterpart whereas all the peaks of exfoliated h-BN nanosheets demonstrate relatively reduced intensity. Furthermore, the (004) and (002) peaks remain suggesting the short range order of the BN crystals in *c*-direction mostly intact.^40^ Of more interest is the intensity ratio of the (004) and (100) planes because it is unusually intensive for all the exfoliated samples. Fig. 3b illustrates the intensity ratio of *I*(004)/*I*(100) of exfoliated BNNSs with different nanosheet length after centrifugation selection. *I*(004)/*I*(100) is 0.7 for the bulk pristine of h-BN powder, whereas it is increased gradually from 0.9 to 2.3 for the exfoliated BNNSs with nanosheet length of 1–2.2 μm as guided by the solid line in Fig. 3b. In fact, the exfoliation process occurs along the (002) plane, leading to the expose of the (002) planes of as-exfoliated BNNSs. As the exfoliated BNNSs preferentially orientate into substrates with the (002) basal plane parallel to the substrate surface, this enhanced intensity ratio of *I*(004)/*I*(100) in XRD measurement can be attributed to both the reduced layer thickness and the large diameter to thickness ratio of the nanosheets.^41^ The longitudinal layer thickness of these nanosheets after each high-rate centrifugation process is constantly in the range of 1–6 atomic layer as confirmed by AFM, thus such significant change in intensity ratio of *I*(004)/*I*(100) is exclusively ascribe to the variation of the lateral nanosheet length, which will be further demonstrated as the following.

**Fig. 4:**
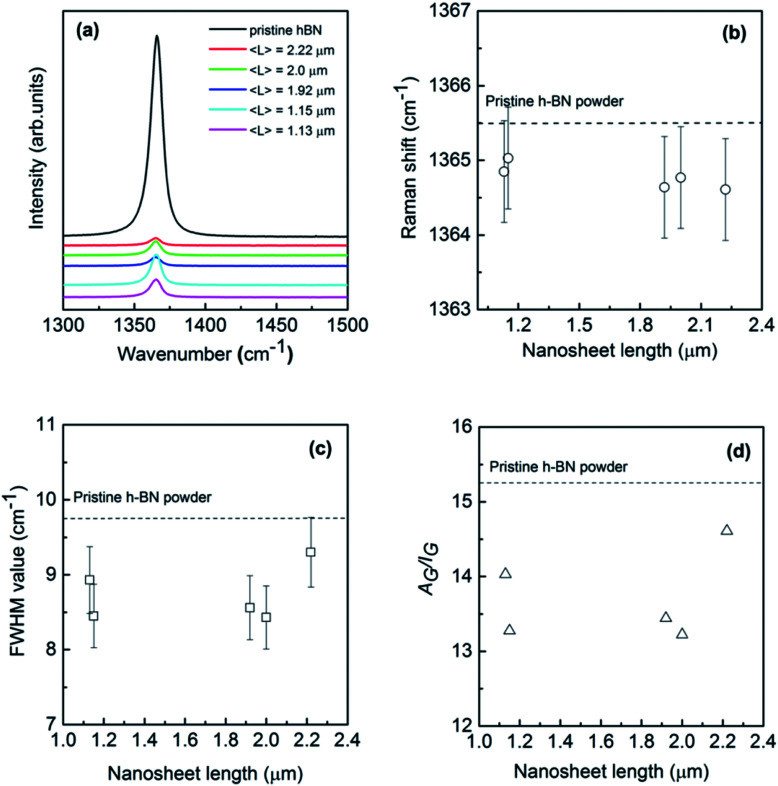
(a) Typical Raman spectra of exfoliated BNNSs with different lateral nanosheet length. (b–d) Raman shift, full width at half maximum (FWHM) value and the ratio of area/intensity of E_2g_ mode of the exfoliated BNNSs as a function of lateral nanosheet length. The dash line is added as a reference of the typical pristine bulk h-BN powder.

Raman scattering is known to be a sensitive tool for the detection of disorder within a sample induced by, *e.g.*, impurities, defects, and size-induced effects of the system under investigation. Therefore, it is considered to be quite effective in studying the graphene in which there are generally D, G, and 2D characteristic bands around 1350, 1580 and 2700 cm^−1^ and the longitude and lateral sizes can be unambiguously determined by the Raman intensity ratio between G and D (2D) bands.^33,34^ However, BN nanosheets do not exhibit a Raman D band due to the lack of Kohn anomaly, leaving difficulties in extracting the size information through similar procedure. Fig. 4a shows Raman spectra of the pristine bulk h-BN powder and exfoliated BNNSs with different lengths excitation by a 514 nm laser beam at room temperature. Bulk h-BN represents a unique prominent G band centered at around 1365.5 cm^−1^ that can be attributed to E_2g_ phonon mode at the Brillouin zone center.^9,42^ When compared to the bulk powder, the nanosheet sample reveals a much lower peak intensity resulting from the reduction of layer thickness and defects created due to exfoliation. Furthermore, the peak position shifts towards low wavenumber direction for about 1 cm^−1^, exiguous as compared to the graphene case where generally a red-shift of ∼20 cm^−1^ can be found.^43^ The red-shift of Raman G bands in thin h-BN atomic layers might be caused by several reasons: (1) heating effect from a laser beam; (2) the interaction between neighbouring layers in bi-layer or few-layer BN nanosheets due to a weak elongation of the B–N bonds;^9^ (3) strain induced by the substrate since the Raman measurements have been executed on SiO_2_/Si substrates.^44,45^ Laser-induced local heating is unlikely since non-resonant Raman spectroscopy was used in this studies.^46^ Furthermore, changing the laser power did not result in any noticeable shift in the peak position. In addition, strain induced by the uneven substrate surfaces generally results in a blue shift of G band to some extent.^35^

The G band is associated with the vibration of in-plane B–N relative motion of hexagonal ring, consequently sensitive to the any defects occurring in the ring. In order to derive at a more quantitative picture of these exfoliated h-BN nanosheets depending on the sheet length, Lorentzian line shapes were attributed to the experimental data. Small frequency shifts and full width at half maximum (FWHM) value of this mode *versus* nanosheet length have been summarized in Fig. 4b and c. As can be seen in Fig. 4b, the G bands of exfoliated BNNSs remain approximately constant as the nanosheet length *L* increases from 1 to 2.4 μm, though slightly shifting to the downward wavenumber directions simultaneously. This indicates that for larger crystals (longitude thickness of 1–6 atomic layers and lateral length of 1–2 μm) the B–N bonds are still sp^2^ kind with no significant strain in the bonds. On the other hand, peak width of Raman band is often considered as a measure for defects in the crystals. Previous reports in graphene demonstrated two main types of defects: body defects such as point defects on the basal plane and edge defects unavoidably during the exfoliation process. Fig. 4c shows that FWHM of exfoliated BNNSs is estimated as between ∼8 to 9 cm^−1^, yet much less than that of the earlier reported BNNSs commonly in the range of ten or several tens of wavenumber.^21,42,47^ Such narrowed FWHM of E_2g_ peak probably arises from lower defects induced by the reduced exfoliation temperature compared to the previously report.^19^ The G band FWHM of all the nanosheets slightly increases as the nanosheet length *L* increases, however the data is much fluctuating to conclude. As the absolute Raman signal intensity depends on many factors, thus, instead of the intensity or the width of the band, the integrated area (*A*) divided by the intensity (*I*) may be more intuitive to deliver information on quality of these nanosheets. As shown in Fig. 4d, *A*_G_/*I*_G_ presents relatively irregular fluctuation with the nanosheet length increases, consistent with the behavior for FWHM of G band. Therefore, Raman measurement is indeed beneficial to confirm the size of graphene and other 2D materials, however, it is not sensitive enough to determine the longitudinal thickness of the exfoliated h-BN atomic layers,^35^ or the lateral length in the present work.

**Fig. 5:**
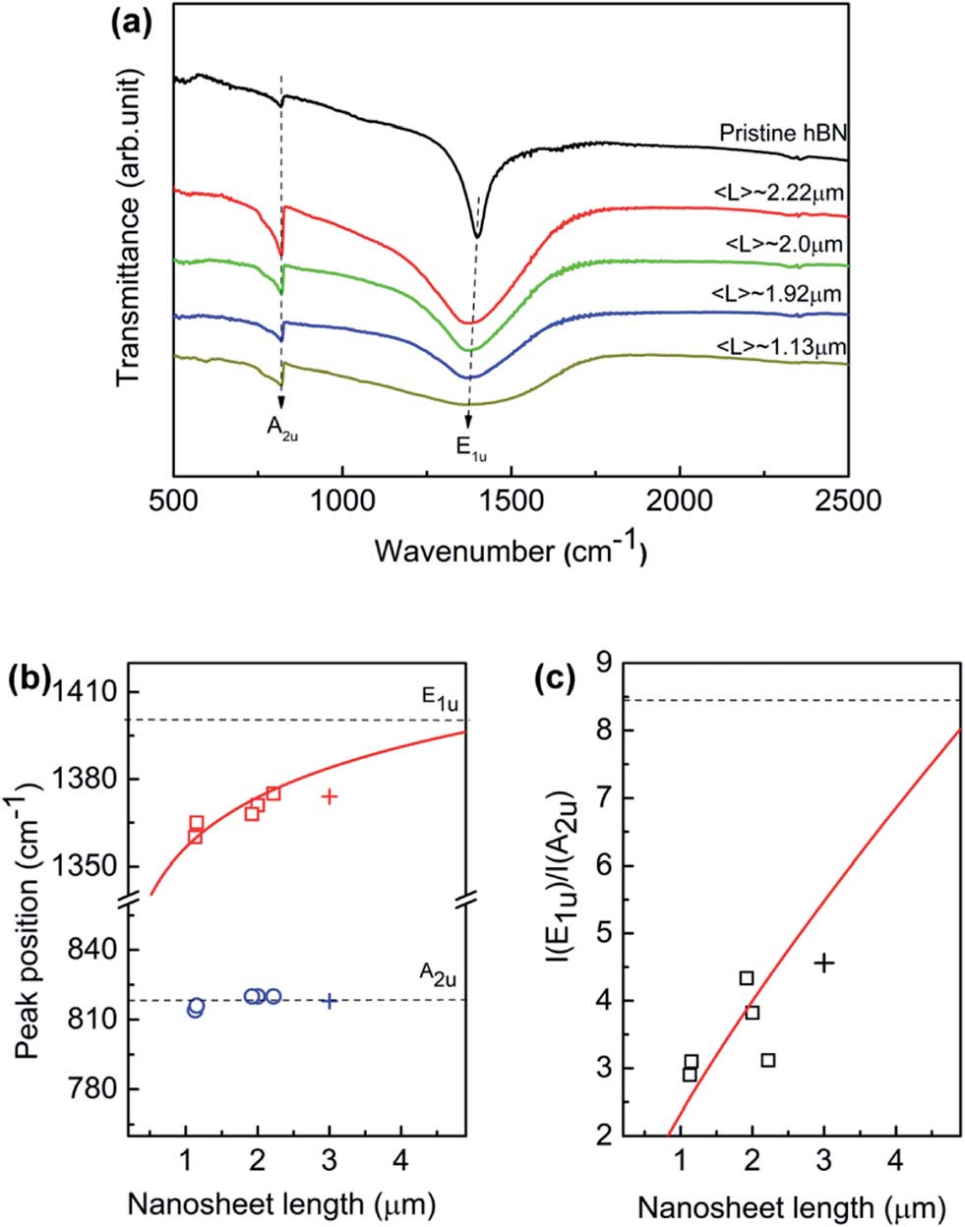
(a) Typical FTIR spectra for the exfoliated BNNSs after size selection with different lateral nanosheet length. (b) The IR frequency of the in-plane E_1u_ and out-of-plane A_2u_ phonon modes as a function of lateral nanosheet length. (c) The intensity ratio of E_1u_ to A_2u_ as a function of lateral nanosheet length. The red solid lines suggesting power laws of *L*. The dash lines in (b) and (c) suggest the pristine bulk h-BN powder. “+” an exfoliated BNNS sample solely sorted with low-rate centrifugation of 3000 rpm.

Fig. 5a displays the FTIR spectra of the exfoliated h-BN nanosheets at room temperature over 400–4000 cm^−1^. The nanosheets were prepared by dispersed in KBr powers to rule out the influence of the substrate. The absorption signal is solely governed by two IR-active phonon peaks, centering at around 1380 and 818 cm^−1^, respectively. The high-frequency E_1u_ phonon peak is ascribed to in-plane oscillation of B and N atoms within the basal plane, while the low-frequency A_2u_ phonon peak is associated with the vibration normal to the basal BN plane. For the normal incident light, only in-plane E_1u_ phonon mode should be present if the BN hexagonal basal plane is perfectly perpendicular to the IR beam. The randomly orientation of BN nanosheets in KBr tablet leads to the presence of out-of-plane A_2u_ mode. As the nanosheet length decreases, the intensities of both E_1u_ and A_2u_ peak decrease; while E_1u_ peak significantly shifts towards to lower wavenumber direction and A_2u_ is unaffected. Generally such red shift of the IR active mode reveals a lower force constant and relaxation between B and N atoms in the planar network. However, the phonon behavior suggests that the lateral size induces differently in the local interaction with the incident IR resonances at nanoscale.

The quantitative relation between the fundamental phonon modes and the lateral nanosheet length is simulated. We derived characteristics of E_1u_ and A_2u_ peaks by executing Lorentzian fitting to the IR curve in Fig. 5a. Fig. 5b illustrates the peak position of E_1u_ and A_2u_ phonon mode as a function of nanosheet length in which the empty symbol and solid line represent the experimental data and fitting results. We can see that E_1u_ mode moves progressively to the higher wavenumber from 1367 to 1380 cm^−1^ as the nanosheet length increases, as well as A_2u_ mode remains invariable independently. The E_1u_ mode frequency increases monotonically as the nanosheet length *L* increases, which is found obeying a power law of 0.02 of nanosheet length (∼*L*^0.02^). Besides, as the nanosheet length increases, the frequency is approximately approaching to the bulk E_1u_ mode in the present work. h-BN is a natural hyperbolic van der Waals material possessing in-plane and out-of-plane components of the dielectric tensor having the opposite signs, giving rise to natural optical anisotropy.^14,48^ The response to the lateral length *L* of the phonon activities is probably associated with the strong anisotropy in the in-plane and out-of-plane axes. Recently, BN nanoribbons have been found that the band gap is dominated by the edge states and it decreases solely with the increasing ribbon width.^49^ The monotonic increase of the E_1u_ phonon frequency implies that the defect-induced distortion in the in-plane mode is pushed up with the increasing lateral length. When the nanosheet length *L* increases, the area of edge increases. Also, the defects are accumulated preferentially near/at the edge area of h-BN sheet according to the theoretical and experimental results.^50,51^ On the other hand, the defects along the out-of-plane axis are approximately invariant since the thickness of the nanosheet fixes. This anisotropy of the defects along the in-plane and out-of-plane is considered of as the origin of the monotonic increases of E_1u_ phonon frequency with increasing nanosheet length *L*. Indeed, it has been found that defects are much higher near the edges than in the interior of the monolayer tungsten disulfide.^52^ Thus, the defect-induced lattice strain may be one possible reason for the phonon frequency shifts as the nanosheet length *L* increases since more relaxed structure should be formed by lower defect density in the interior. In addition, the intensity ratio *I*(E_1u_)/*I*(A_2u_) as a function of nanosheet is plotted as shown in Fig. 5c. The ratio increases in proportion to nanosheet length, as fitted by the solid line based on a power law of *L*^0.78^. It is important to point out that the 3 μm BNNS sample was prepared separately and only sorted with low-rate centrifugation (3000 rpm) as illustrated by “+”, so that it differs from the previous samples since the thicker sheets would be expected at much lower rotation rates. The size changes in both lateral and longitudinal dimension, which probably explains the slightly deviation from the fitting line. The correlation of the anisotropic dependence of these phonon modes on the lateral size is still under research. Nevertheless, the FTIR results provide a good prediction of description of lateral length to the in-plane E_1u_ mode, indicating the E_1u_ peak is possibly an alternative measure for the lateral size aside SEM or TEM.

## 4. Conclusions

To summarize, we have propose a simple and effective centrifugation approach to rapidly select chemically exfoliated BNNSs according to their average lateral size. Centrifugation at higher rates results in smaller h-BN nanosheets being separated. In this case, the exfoliated h-BN nanosheets with thickness of 1–6 atomic layer and lateral length of 1–3 μm can be selected according to the engaged centrifugation rate. The size dependent Raman suggests that the E_2g_ mode of BN is not sensitive enough to determine the longitudinal thickness^35^ or lateral length this work. Interestingly, for FTIR measurements, the in-plane E_1u_ phonon mode and the intensity ratio *I*(E_1u_)/*I*(A_2u_) is found to be intensively dependent on nanosheet length. The implications of the strong anisotropic dependence of E_1u_ and A_2u_ phonon frequency on lateral size of nanosheet are restricted to different defect density between in-plane and out-of-plane axes. Therefore, these results provide a possible spectroscopic measure of lateral length of BNNSs prepared not only by chemical exfoliation in present work but also in other form of liquid dispersions.

## Conflicts of interest

There are no conflicts of interest to declare.

## Supplementary Material

RA-008-C7RA12872J-s001
