# Supplementary material for: Lateral size selection of liquid exfoliated hexagonal boron nitride nanosheets
Source: RSC Adv. 2018 Feb 5;8(11):5976–83. doi: 10.1039/c7ra12872j (PMC9078175; doi:10.1039/c7ra12872j)
Supplement: RA-008-C7RA12872J-s001 [file RA-008-C7RA12872J-s001.pdf]

Electronic Supplementary Material (ESI) for RSC Advances.
This journal is © The Royal Society of Chemistry 2018

Supporting Information

Manuscript ID: RA-ART-11-2017-012872

TITLE: Lateral Size Selection of Liquid Exfoliated Hexagonal Boron Nitride Nanosheets

The exfoliated h-BN nanosheets after high-rate centrifugation have been measured by TEM. Fig.S1(a) presents a typical BNNSs sample dispersed on the carbon film where monolayer and few-layer nanosheets stack together. The top layer is nearly transparent to the electron beam so that the bottom layer distinctly appears. Fig.S1(b) represents the atomic structure where a typical hexagonal lattice structure of a monolayer BNNS is demonstrated in which the d spacing of 0.33 nm is measured. This value is quite approaching the d(002) of h-BN. Furthermore, the inset shows the relevant selected area electron diffraction (SAED) of this nanosheet taken with electron beam along [001] zone axis, perpendicular to the sheet surface. It marks the singly crystallinity in nature and the hexagonal structure of the exfoliated sample.

We measured the edges of these sheets, since the edges of the individual sheets are almost always distinguishable in TEM images. We designate the sheet thickness as the layer number per sheet. By carefully counting the layer edges, it is possible to measure the number of layers per sheet. Certainly for some specially cases, it is extremely difficult to recognize the edges. Thus it is expected the errors involved to be random and can be cancelled out when statistical results are analyzed in histogram. On the other hand, we note that for the higher rate centrifugation, the amount of selected sheets are reduced so that the TEM grids are sparsely coated by nanosheets, making them difficult searched by TEM.

Fig.S2 illustrate TEM images of typical exfoliated h-BN nanosheets after centrifugation process and each corresponding histogram of mean layer number per sheet. As can be observed, the thickness of these nanosheets after high-rate centrifugation process ranges from monolayer to multiple layers. It is noticeable that there are very thick sheets on rare occasions (for example, 12 layers observed for sample after 5000/18000rpm centrifugation). We use Gaussian distribution function to extract mean values for these data. It is failed to extract mean value for Fig. S2(b) and S2(j) probably due to the lack of data points or wide spread of size distributions. Nevertheless, it can be estimated that the mean thickness of these exfoliated h-BN

nanosheets consist of less than six layers after high-rate centrifugation process. Furthermore, their thickness seems not sensitive to the high-rate centrifugation process.

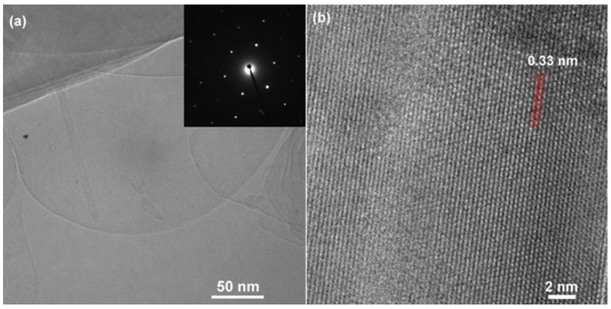

Figure S1 (a) the representative TEM image showing monolayer and few layered thin BNNSs, (b) high resolution TEM image depicting the lattice structure of the monolayer and the inset in (a) illustrating the SAED pattern.

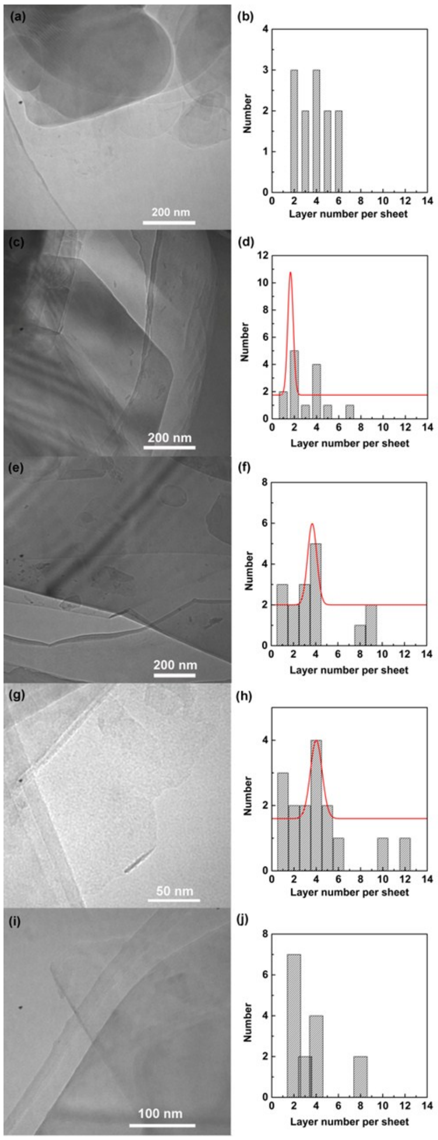

Figure S2 TEM images of typical exfoliated h-BN nanosheets after centrifugation process of 3000/16000rpm (a), 3000/18000rpm (c), 3000/20000rpm (e), 5000/18000rpm (g) and 5000/20000rpm (i) and their corresponding histogram of mean layer number per sheet.
